# *Enterococcus faecalis* Translocation in Sepsis: Fibrinolysis and Mitochondrial Dysfunction Drive Lung Injury

**DOI:** 10.1111/jcmm.70937

**Published:** 2025-11-11

**Authors:** Chenfei Wang, Dan Lv, Yuan Gao, Xinhui Xu, Changqing Zhu, Song Zhang, Keji Zhang

**Affiliations:** ^1^ Department of Emergency, Ren Ji Hospital, School of Medicine Shanghai Jiao Tong University Shanghai China; ^2^ Department of Critical Care Medicine, Ren Ji Hospital, School of Medicine Shanghai Jiao Tong University Shanghai China

**Keywords:** *Enterococcus faecalis*, fibrinolytic system, gut‐lung axis, lung injury, mitochondrial dysfunction, sepsis

## Abstract

Sepsis frequently progresses to acute lung injury (ALI), characterised by inflammation, extracellular matrix degradation, and mitochondrial dysfunction. This study identifies *Enterococcus faecalis* as a gut‐derived bacterium that exploits the host fibrinolytic system for pulmonary translocation, resulting in mitochondrial damage and exacerbating lung injury. Utilising the cecal ligation and puncture (CLP) mouse model combined with *E. faecalis* pulmonary infection, we demonstrated that *E. faecalis* exacerbates lung injury by activating fibrinolysis, disrupting intestinal barrier integrity, and impairing mitochondrial function. Key findings include elevated plasmin activity, increased fibrin degradation products (FDP), and reduced expression of tight junction proteins ZO‐1 and occludin. Mitochondrial dysfunction was confirmed by disrupted ultrastructure, impaired ATP synthesis, and increased ROS levels. Histological analyses revealed severe alveolar damage, neutrophil infiltration, and edema. Treatment with the fibrinolysis inhibitor aminocaproic acid or the mitochondrial protector MitoTEMPO alleviated fibrinolytic activity, preserved mitochondrial function, and reduced lung damage. Notably, combination therapy showed the most significant protective effects, improving lung histology and decreasing inflammation markers. This study provides novel insights into sepsis‐induced lung injury, highlighting *E. faecalis* and the fibrinolytic system as potential therapeutic targets.

**Abbreviations**
AVMA: American Veterinary Medical Association
CLP: cecal ligation and puncture
DAPI: 4′,6‐diamidino‐2‐phenylindole
ELISA: Enzyme‐Linked Immunosorbent Assay
HE: haematoxylin and eosin
IECs: intestinal epithelial cells
PAS: periodic acid‐Schiff
ROS: reactive oxygen species
SD: standard deviation

## 1 Introduction

Sepsis is a life‐threatening syndrome characterised by systemic inflammation and multi‐organ failure [1, 2, 3], with acute lung injury (ALI) being a leading cause of mortality in severe cases [4, 5]. ALI is hallmarked by alveolar damage, extracellular matrix (ECM) degradation, and disrupted mitochondrial function; however, the exact mechanisms driving lung injury during sepsis remain incompletely understood. Recent studies have highlighted the gut‐lung axis as a critical pathway in sepsis pathophysiology, where intestinal barrier dysfunction facilitates the translocation of intestinal microbes to the lungs, triggering severe inflammatory responses [6, 7]. Although microbial translocation is a driver of sepsis‐related lung damage [8], research on specific gut bacterial species involved in sepsis‐induced lung injury remains scarce. For example, gram‐negative bacteria (such as *Escherichia coli* ) release endotoxin lipopolysaccharide (LPS), which induces pulmonary inflammatory responses and causes lung injury [9]. Additionally, membrane vesicles (MVs) released by some bacteria like *Pseudomonas aeruginosa* damage endothelial barrier integrity and synergise with inflammatory responses to further exacerbate lung damage [10]. However, the pathways through which these bacteria enter the lungs and exert their effects remain unclear.

*Enterococcus faecalis* ( *E. faecalis* ), a gut‐derived opportunistic pathogen, has been implicated in extraintestinal infections during intestinal barrier disruption. Recent evidence indicates that *E. faecalis* can hijack the host fibrinolytic system to degrade ECM components, such as collagen, enabling tissue invasion and colonisation of distant organs [11]. However, whether *E. faecalis* utilises the fibrinolytic system to invade the lungs, exacerbate mitochondrial dysfunction, and tissue injury during sepsis remains unestablished.

The fibrinolytic system, often dysregulated during sepsis [12, 13, 14, 15, 16], plays a dual role in maintaining vascular integrity and facilitating bacterial dissemination. During microbial invasion, increased fibrin and plasmin production promote thrombus formation and induce inflammatory responses, thereby limiting infection spread [12]. However, in sepsis patients, excessive and dysregulated activation of coagulation pathways leads to overproduction of thrombin, triggering disseminated intravascular coagulation (DIC). This induces systemic inflammation, which finally contributes to organ failure [17]. Evidence suggests that binding and activating plasminogen enhance microbial translocation capability [18, 19], but no direct evidence currently establishes the impact of the fibrinolytic system on microbial translocation in sepsis.

This study identifies *E. faecalis* as a key driver of sepsis‐induced ALI, demonstrating its exploitation of the host fibrinolytic system for the gut‐to‐lung migration, where it induces mitochondrial damage and amplifies pulmonary injury. By investigating the interplay between microbial translocation, fibrinolytic activation, and mitochondrial dysfunction, this work provides novel mechanistic insights into sepsis‐induced lung injury. These findings not only position *E. faecalis* as a potential therapeutic target but also emphasise the critical role of the gut‐lung axis in sepsis pathophysiology.

## 2 Materials and Methods

### 2.1 Animal Model

All animal procedures were conducted in strict adherence to the guidelines for the care and use of laboratory animals. Adult male C57BL/6 mice (8–12 weeks old, 20‐25 g) were obtained from the Shanghai SLAC Laboratory Animal Co. Ltd. (Shanghai, China). Mice were housed under controlled conditions with a 12‐h light/dark cycle, and provided with ad libitum access to food and water. After 1 week of acclimatisation, sepsis‐induced acute lung injury was established using the cecal ligation and puncture (CLP) model [20]. All animal experiments were approved by the Ethics Committee of Experimental Animal Welfare of Renji Hospital Affiliated to Shanghai Jiaotong University.

Surgical procedures were performed under sodium pentobarbital anaesthesia (50 mg/kg, intraperitoneal injection). A 2‐cm midline abdominal incision was made to expose the cecum, which was carefully dissected from the mesenteric attachment. The cecum was tightly ligated at 1/2 of the distal end with sterile 4‐gauge silk thread, and punctured at the center of the distal end with a sterile 7‐gauge needle. A small amount of intestinal contents was gently squeezed out, minimising the damage to blood vessels. The cecum was then returned to the abdominal cavity, and the incision was sutured closed. Sham‐operated controls underwent identical procedures excluding ligation and puncture.

### 2.2 Drug Administration

*E. faecalis* (10^9^ CFU of a live preparation, Symbioflor1) was suspended in 35 μL of excipient solution and 15 μL of Ora‐Sweet (Paddock Laboratories), administered orally via micropipette. Fibrinolysis inhibitor (ε‐aminocaproic acid, 100 mg/kg) was administered via intraperitoneal injection [21]. Mitochondrial protectants (e.g., MitoTEMPO) were administered, with Mito‐Paraquat stock solution prepared in ethanol and diluted in PBS. Adult mice were intraperitoneally injected with 10 μM at a dose of 0.1 μg/kg body weight.

### 2.3 Sample Collection

At 24 h post sepsis induction and interventions, mice were euthanised in accordance with the recommendations of the *American Veterinary Medical Association (AVMA) Guidelines for the Euthanasia of Animals*. Intestinal tissues (specifically the jejunum) were surgically dissected. Samples were then fixed in 4% paraformaldehyde for 24 h at 4°C, followed by dehydration, embedding in paraffin, and sectioning at 5 μm thickness for subsequent analyses.

### 2.4 Histopathological Analysis

Lung tissues were fixed in 4% paraformaldehyde (PFA) for 24 h, followed by sequential treatments with 70% PFA for 2 h, 80% PFA overnight, 90% PFA for 2 h, and 100% PFA for two‐hourly intervals at room temperature. The tissues were then subjected to ethanol dehydration. Paraffin‐embedded tissues were sectioned into 4‐μm slices and stained with haematoxylin and eosin (H&E) and Masson's trichrome according to standard protocols. The sections were evaluated by light microscopy, and images were captured. The severity of pulmonary fibrosis (PF) was assessed based on the criteria described by Szapiel et al. [22].

### 2.5 Immunofluorescence Staining

The frozen tissue sections (5 μm thick) were fixed in acetone for 10 min at −20°C, followed by blocking with 5% bovine serum albumin in PBS for 1 h at room temperature to prevent nonspecific binding. Sections were then incubated overnight at 4°C with primary antibodies against Claudin‐1, Occludin and ZO‐1 diluted in blocking buffer. After washing with PBS, the sections were incubated for 1 h at room temperature in the dark with secondary antibodies: goat anti‐rabbit IgG conjugated with Alexa Fluor 488 or Texas Red (Invitrogen). Nuclei were counterstained with 4′,6‐diamidino‐2‐phenylindole (DAPI). Fluorescence intensity was analysed using ImageJ software (v1.8.0).

### 2.6 Intestinal Permeability Assay

Following a 4‐h fast, mice (randomised order) received oral gavage of FITC‐Dextran (600 mg/kg, Sigma‐Aldrich, USA). At 2 h post‐dosing, mice were anaesthetised with isoflurane (induction at 5%, maintenance at 2% isoflurane, with 0.7 L/min N_2_O and 0.3 L/min O_2_). Blood was then collected via retro‐orbital puncture into K3‐EDTA‐coated tubes (Sarstedt, Germany), followed by euthanasia via cervical dislocation. The blood samples were centrifuged at 4°C for 7 min at 8000 g, and the plasma was harvested into clear Eppendorf tubes (Eppendorf AG, Hamburg). Plasma samples from mice treated with PBS were used to establish the standard curve. The concentrations of FITC‐Dextran in the plasma were determined in duplicate using a fluorescence spectrophotometer (SpectraMax M4, Molecular Devices, San Jose, CA, USA) with excitation at 485 nm and emission at 535 nm.

### 2.7 Alkaline Hydrolysis Method

The alkaline hydrolysis method was employed to determine the hydroxyproline (Hyp) concentration in lung tissues and serum. The absorbance of each sample tube was measured using a spectrophotometer (Shanghai Analytical Instrument Co., Shanghai, China). The Hyp concentration (μg/mg) was then calculated as follows [23]:

$$\mathrm{Hyp}=\frac{{\mathrm{Abs}}_{\text{sample}}-{\mathrm{Abs}}_{\text{blank}}}{{\mathrm{Abs}}_{\text{standard}}-{\mathrm{Abs}}_{\text{blank}}}\times C_{\text{standard}}\times \frac{V_{\text{hydrolyzate}}}{w_{\text{tissue}}}$$

*Abs = Absorbance, *C*_standard_ = Hyp standard concentration (5 μg/mL), *V*_hydrolysate_ = Total hydrolysate volume (5 mL), *w*_tissue_ = tissue wet weight (mg).

### 2.8 Reactive Oxygen Species (ROS) Measurement

ROS levels in intestinal tissues were quantified using a ROS test kit (E‐BC‐K138‐F, Elabscience), following the provided instructions. A single cell suspension was prepared from intestinal tissue, to which DCFH‐DA working solution was added. Fluorescence intensity was measured using a microplate reader at λ_ex_ 500 nm/λ_em_ 525 nm.

### 2.9 ATP Concentration Assay

ATP concentrations were measured using a commercial ATP assay kit (e.g., CellTiter‐Glo Luminescent Assay, Promega) according to the manufacturer's protocol. Briefly, 20–50 μL of the supernatant was mixed with the ATP reagent in a 96‐well plate, followed by a 10‐min incubation at room temperature. Luminescence was measured via a microplate reader, with ATP concentrations calculated based on a standard curve. Results were normalised to tissue weight and expressed as μmol/g.

### 2.10 Transmission Electron Microscopy

Mouse intestinal tissues were dehydrated, sliced into ultrathin sections, and stained with 3% uranyl acetate followed by lead citrate. Finally, the samples were observed under an 80 kV transmission electron microscope to analyse morphological changes of mitochondria.

### 2.11 Enzyme‐Linked Immunosorbent Assay (ELISA)

BALF supernatant was obtained as previously described [24]. After retrieval, BALF was placed on ice, centrifuged, and aliquoted; supernatant was stored at −80°C until analysis. The concentrations of fibrin degradation products in the BALF were quantified using ELISA kits (BD Biosciences) according to the manufacturer's protocol. Serum samples were diluted 1:50 and assayed in duplicate. Absorbance was measured at 450 nm using the Promega GloMax Explorer microplate reader.

### 2.12 16S rRNA Sequencing

Fresh mice feces were collected and stored in sterile containers at −80°C. Microbial genomic DNA was extracted from these samples using a DNA extraction kit. The bacterial V4 variable regions of 16S rRNA genes were sequenced. The sequencing results were analysed using Silva and RDP databases.

### 2.13 Quantitative Real‐Time PCR Amplification

Quantitative real‐time polymerase chain reaction (qPCR) quantified *Enterococcus faecalis* , *Staphylococcus aureus* , and *Escherichia coli* in mouse lung tissues. DNA was extracted from lung tissues using the FastDNA spin kit for soil (MP Biomedicals, CA, United States) following the manufacturer's instructions, and was stored at −20°C until analysis. qPCR was performed on an ABI StepOnePlus Real‐Time PCR System (Applied Biosystems, MA, United States) using the TOPreal qPCR 2× PreMIX (SYBR Green with high ROX, Enzynomics). The 20 μL reaction mixture contained 10 μL PreMIX, 1 μL each of forward and reverse primers (10 pmol/μL), and 1 μL of DNA template (12 ng/μL). Amplification involved 95°C for 10 min (initial denaturation), 40 cycles of 95°C for 15 s, 60°C for 1 min, 72°C for 30 s. Melting curve analysis was performed to confirm the specificity of the amplification.

The primers used were:

-
*E. faecalis* : F 5′‐CCG AGT GCT TGC ACT CAA TTG G‐3′, R 5′‐CTC TTA TGC CAT GCG GCA TAA AC‐3′ (137 bp);
-
*S. aureus* : F 5′‐GTT GCT TAG TGT TAA CTT TAG TTG TA‐3′, R 5′‐AAT GTC GCA GGT TCT TTA TGT AAT TT‐3′;
-
*E. coli* : F 5′‐GTT AAT ACC TTT GCT CAT TGA‐3′, R 5′‐ACC AGG GTA TCT AAT CCT GTT‐3′;
- Universal bacteria: F 5′‐ACG TCR TCC MCN CCT TCC TC‐3′, R 5′‐GTG STG CAY GGY YGT CGT CA‐3′.

### 2.14 Statistical Analysis

Data wherever applicable were presented as mean ± standard deviation (SD). Differences between the groups were evaluated using an unpaired two‐tailed Student's t‐test. All statistical analyses were performed using GraphPad Prism 10.1.2. The levels of statistical significance were set at: *, *p* < 0.05; **, *p* < 0.01; ***, *p* < 0.001; ****, *p* < 0.0001.

## 3 Results

### 3.1 Sepsis‐Induced Intestinal Barrier Dysfunction

**FIGURE 1:**
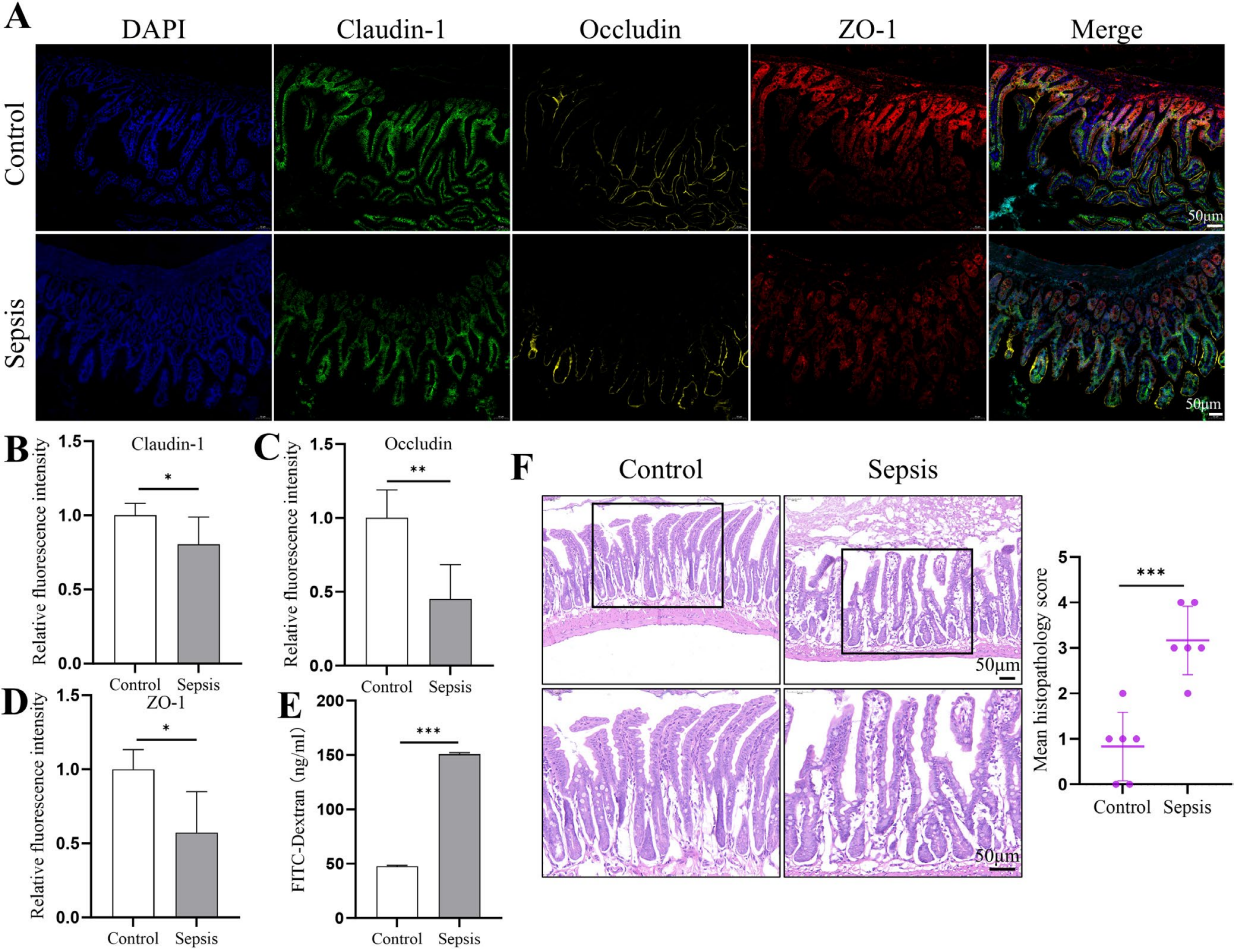
Sepsis‐induced intestinal barrier dysfunction. (A) Immunofluorescence staining of tight junction proteins ZO‐1 (red), occludin (yellow), and claudin‐1 (green) in intestinal tissues. Nuclei were counterstained with DAPI (blue). The sepsis group exhibited reduced and disrupted protein localisation compared to controls. (B–D) Quantification of fluorescence intensity for claudin‐1, occludin, and ZO‐1. Septic mice showed significantly lower levels compared to controls. (E) FITC‐Dextran Permeability Assay revealed increased epithelial permeability in septic mice, indicated by elevated FITC‐Dextran levels in blood. (F) Histological examination (HE staining) showed epithelial exfoliation, villous rupture, and inflammatory infiltration in the sepsis group, with significantly higher pathology scores compared to controls. Data are provided as the mean ± SD (*n* = 6 per group). *p < 0.05, **p < 0.01, ***p < 0.001.

Sepsis significantly disrupted intestinal barrier function, supported by multiple analyses. Immunofluorescence staining (Figure 1A) showed markedly reduced tight junction proteins: ZO‐1 (red), occludin (yellow), and claudin‐1 (green) in the intestinal epithelium of septic mice (DAPI‐counterstained nuclei, blue). The control group displayed a uniform distribution of these proteins at cell borders, whereas the sepsis group exhibited diffuse and irregular patterns. Quantified fluorescence intensity (Figure 1B–D) revealed significant reductions in occludin and ZO‐1 levels in septic mice compared to controls. Although the reduction in claudin‐1 levels was modest, it nevertheless reached statistical significance.

Epithelial permeability assessed using the FITC‐Dextran Permeability Assay (Figure 1E) demonstrated significantly increased serum levels in septic mice, indicating compromised barrier integrity. Histological examination (Figure 1F) using H&E staining corroborated these findings: controls displayed intact intestinal epithelium with structured villi and minimal inflammation; septic mice exhibited epithelial exfoliation, villous rupture, and inflammatory infiltration, with significantly higher pathology scores.

These results collectively confirm severe disruption of intestinal barrier integrity during sepsis, as evidenced by loss of tight junction protein expression, increased epithelial permeability, and pronounced histological damage.

### 3.2 Sepsis‐Induced Lung Injury

**FIGURE 2:**
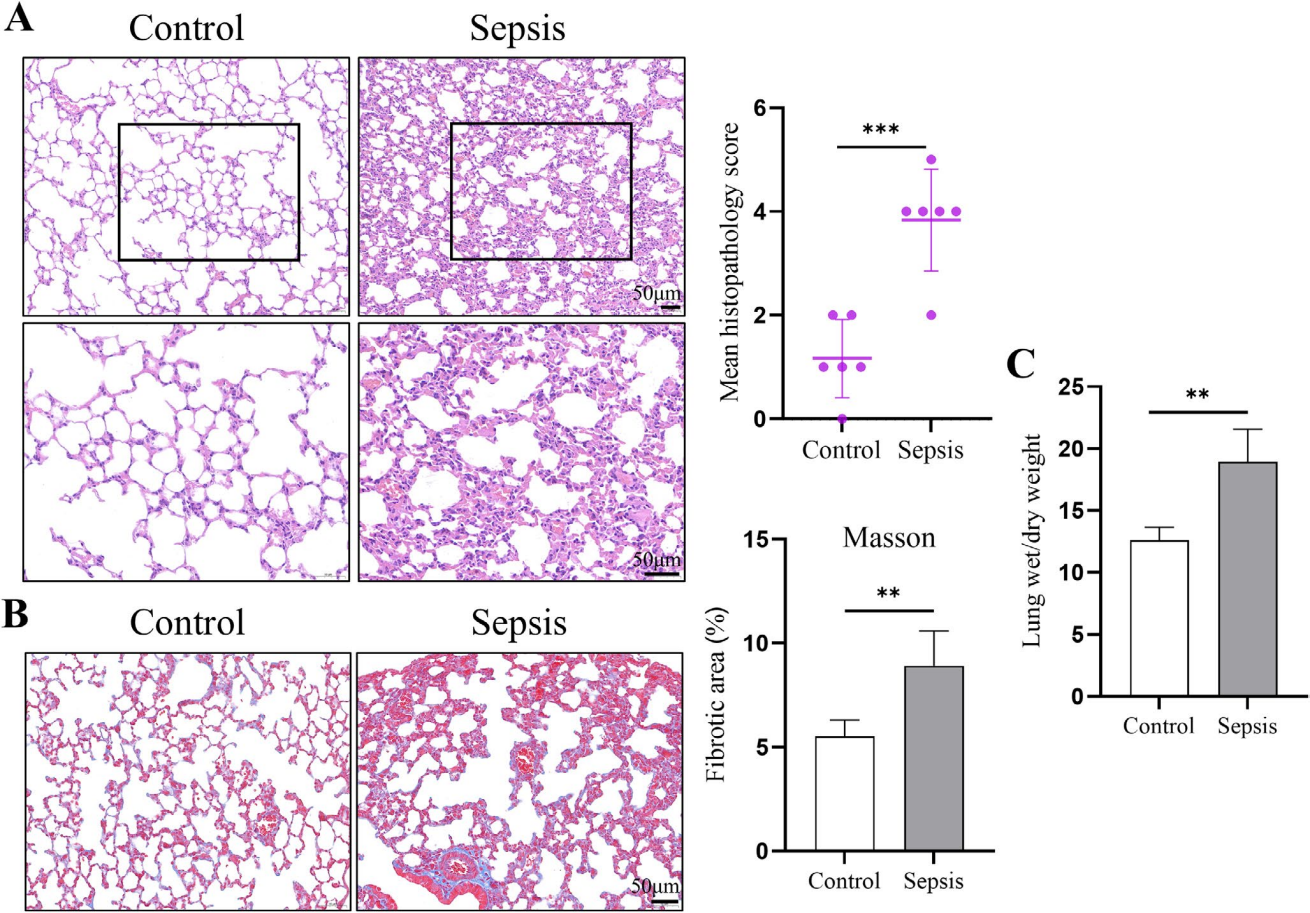
Sepsis‐induced lung injury. (A) Representative HE staining images of lung tissues. Septic mice showed alveolar collapse, haemorrhage, and inflammatory infiltration. The pathology scores were significantly higher in the sepsis group (inset graph). (B) Masson staining demonstrated increased collagen deposition in septic lung tissues, indicative of early fibrosis. Quantification confirmed significantly larger fibrotic areas in the sepsis group (inset graph). (C) Wet‐to‐dry weight ratios showed increased pulmonary edema in septic mice, reflecting severe lung injury Data are provided as the mean ± SD (*n* = 6 per group). **p < 0.01, ***p < 0.001.

Sepsis also led to significant lung injury, supported by multiple assessments. H&E staining (Figure 2A) revealed marked pathological changes in septic mice, including alveolar collapse, haemorrhage, and inflammatory cell infiltration. Corresponding pathology score analysis (inset graph) confirmed significantly higher scores in the sepsis group compared to the control group.

Masson staining (Figure 2B) showed increased collagen deposition in septic lungs, indicative of early pulmonary fibrosis. Quantification of fibrosis area (inset graph) revealed a significant elevation in the sepsis group compared to controls. Furthermore, the wet‐to‐dry weight ratio (Figure 2C), a marker of pulmonary edema, rose significantly in septic mice, reflecting severe lung injury. These findings collectively underscore the substantial lung damage and fibrosis induced by sepsis.

### 3.3 Microbiome Changes in Sepsis

**FIGURE 3:**
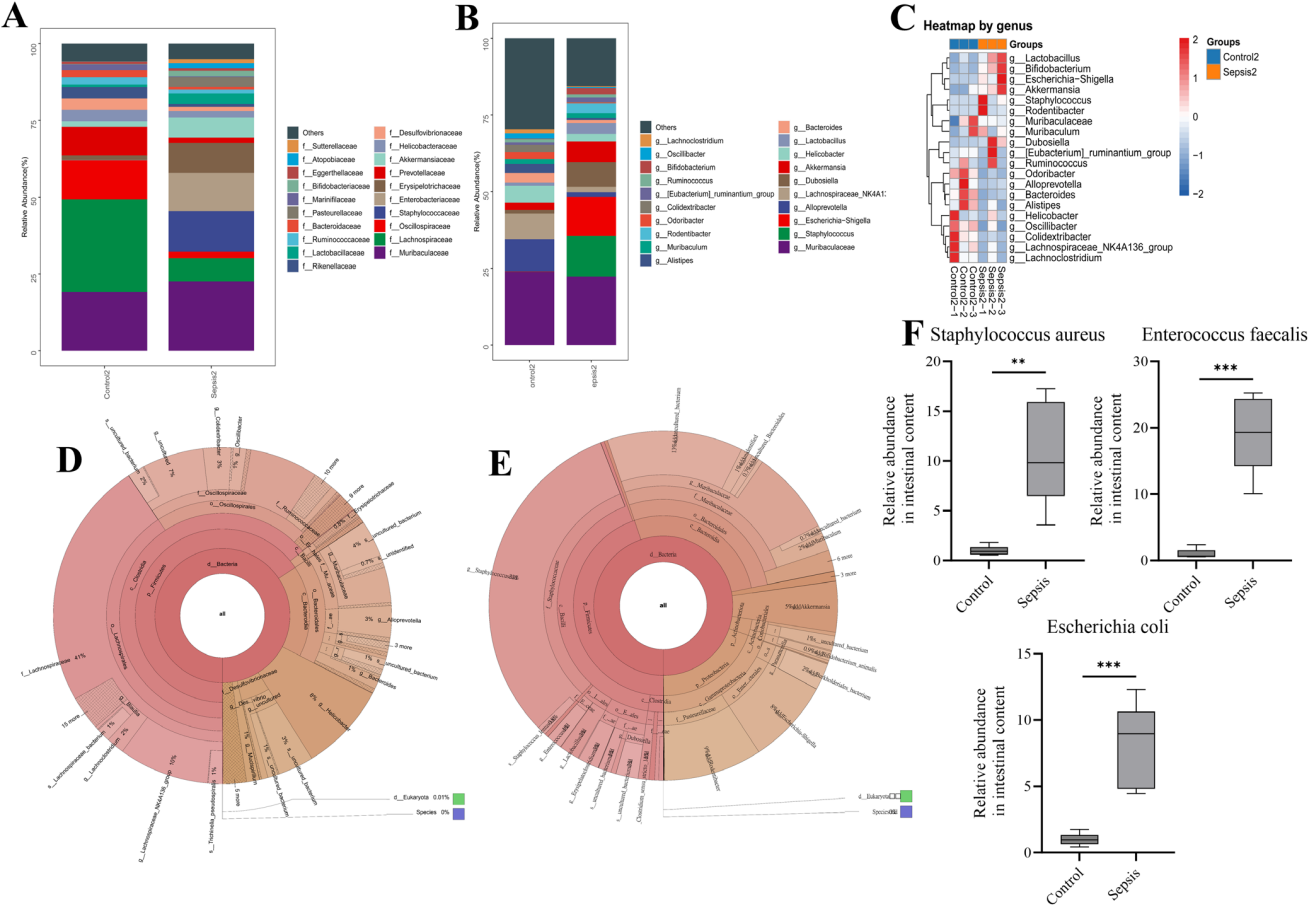
Microbiome changes in sepsis. (A) Relative abundance of pulmonary bacterial families. Septic mice exhibited increased abundance of Staphylococcaceae and Enterobacteriaceae compared to controls. (B) Genus‐level analysis showed increased relative abundance of Staphylococcus and Escherichia−Shigella in the sepsis group, while Muribaculaceae and Alloprevotella predominated in controls. (C) Heatmap showing distinct bacterial composition between sepsis and control groups. (D, E) Krona plots demonstrated a decrease in Firmicutes and an increase in Bacteroidia in septic lungs. (F) Quantitative analysis revealed a significant increase in *Enterococcus faecalis* ( *E. faecalis* ), *Staphylococcus aureus* , and *Escherichia coli* in septic lungs, with *E. faecalis* showing the highest relative abundance Data are provided as the mean ± SD (*n* = 6 per group). **p < 0.01, ***p < 0.001, ****p < 0.0001.

Pulmonary microbiota of mice was profiled by bacterial 16S ribosomal RNA gene sequencing of extracted lung tissues. At the family level, the dominant bacteria in the control group included *Muribaculaceae*, *Lachnospiraceae*, and *Oscillospiraceae*, while the sepsis group showed a significant increase in the relative abundance of *Staphylococcaceae* and *Enterobacteriaceae* (Figure 3A). At the genus level, *Muribaculaceae*, *Alloprevotella*, and *Lachnospiraceae* were predominant in the control group. Septic mice exhibited markedly elevated *Staphylococcus* and *Escherichia − Shigella* (Figure 3B).

Heatmaps revealed the differences in bacterial abundance and composition between the control and sepsis groups (Figure 3C). Among them, increased *Lactobacillus* [25], *Bifidobacterium* [25], and *Akkermansia* [26] typically associate with anti‐inflammatory effects. Krona analysis demonstrated decreased *Firmicutes* but increased *Bacteroidia* in the sepsis group compared to controls (Figure 3D,E). Additionally, quantitative analysis of key bacterial species (Figure 3F) showed a significant increase in the relative abundances of *Enterococcus faecalis* , *Staphylococcus aureus* , and *Escherichia coli* in septic lungs, with *E. faecalis* exhibiting the highest relative abundance. Notably, *Staphylococcus* contributes to lung injury [27]; *Escherichia coli* and *Escherichia‐Shigella* levels also correlate with sepsis‐induced lung injury [28, 29]. This finding highlights the potential role of *E. faecalis* in sepsis‐induced pulmonary complications, supporting its selection for further mechanistic investigations.

### 3.4 Fibrinolytic Activation, Collagen Degradation, and Mitochondrial Dysfunction Exacerbated by *Enterococcus faecalis* in Septic Lungs

**FIGURE 4:**
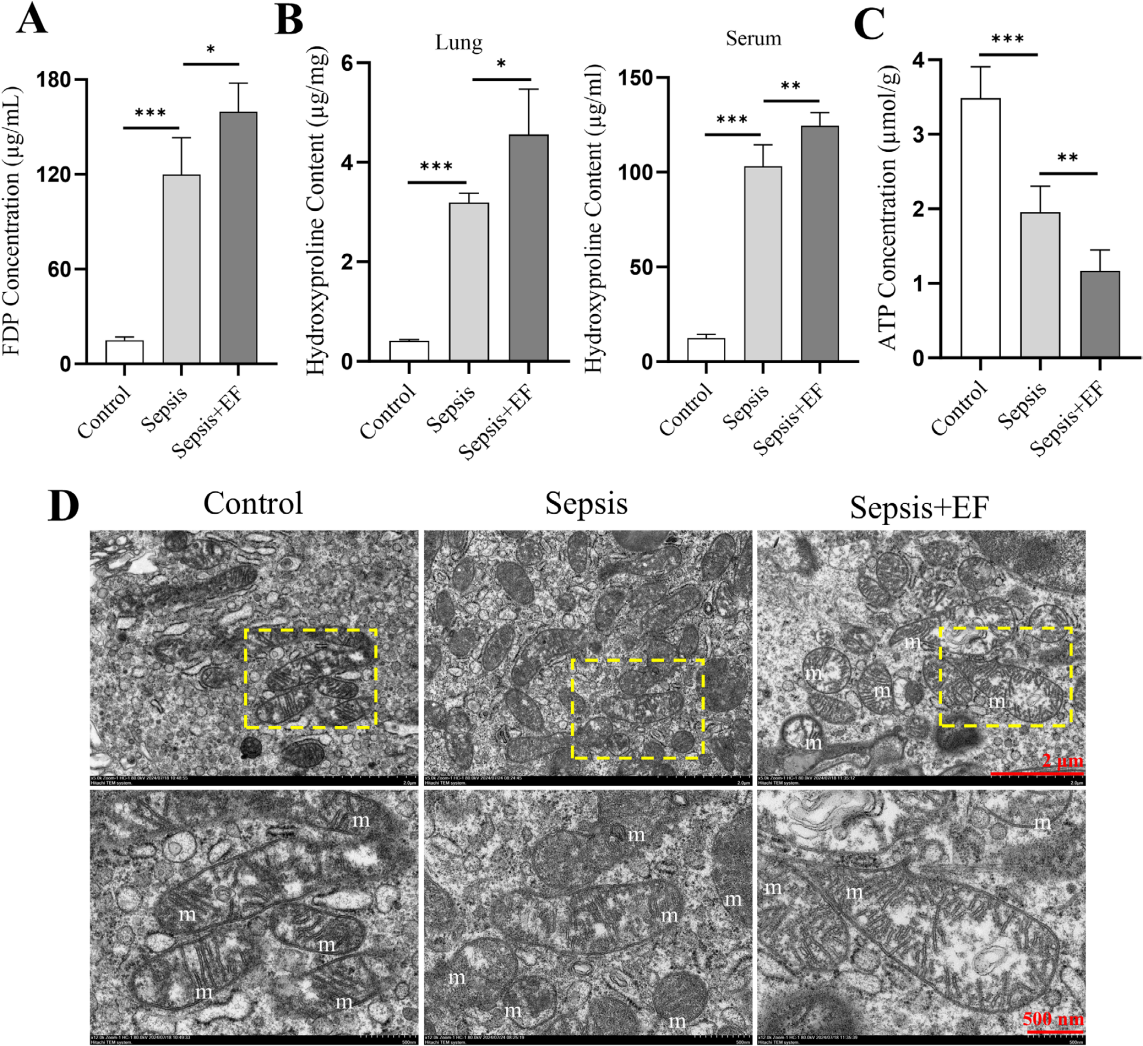
Fibrinolytic activation, collagen degradation, and mitochondrial dysfunction exacerbated by *E. faecalis* in septic lungs. (A) ELISA analysis demonstrated elevated fibrin degradation product (FDP) levels in septic mice, with further increases in the sepsis + *E. faecalis* group, indicating enhanced fibrinolytic activation. (B) Hydroxyproline content analysis revealed significant collagen degradation in lung and blood tissues, with the most pronounced effect observed in the sepsis + *E. faecalis* group. (C) ATP levels were significantly reduced in septic lungs, with further decreases in the sepsis + *E. faecalis* group, indicating severe mitochondrial dysfunction. (D) Ultrastructural analysis via electron microscopy showed mitochondrial swelling, rupture, and cristae loss in septic lungs, with the most severe damage observed in the sepsis + *E. faecalis* group. Data are presented as mean ± SD (*n* = 6 per group). *p < 0.05, **p < 0.01, ***p < 0.001, ****p < 0.0001.

Sepsis significantly activated the fibrinolytic system and induced collagen degradation. *E. faecalis* infection further amplified these effects. ELISA analysis revealed elevated fibrin degradation product (FDP) levels in the sepsis group compared to controls, with the highest levels in the sepsis + *E. faecalis* group (Figure 4A). Similarly, hydroxyproline content, a marker of collagen degradation, was significantly increased in lung and blood tissues of septic mice, peaking in the sepsis + *E. faecalis* group (Figure 4B). These findings highlight *E. faecalis* as a potent driver of fibrinolytic activation and extracellular matrix remodelling in sepsis.

In addition, *E. faecalis* infection exacerbated mitochondrial dysfunction under septic conditions. ATP levels were significantly reduced in septic lungs, indicating impaired mitochondrial energy production; the most severe decline occurred in the sepsis + *E. faecalis* group (Figure 4C). Ultrastructural analysis by electron microscopy revealed pronounced mitochondrial damage in septic lungs, including swelling, rupture, and cristae loss, with the most severe alterations seen in the sepsis + *E. faecalis* group (Figure 4D). These results demonstrate that *E. faecalis* exacerbates fibrinolytic activation, collagen degradation, and mitochondrial dysfunction, collectively contributing to the progression of sepsis‐induced acute lung injury.

### 3.5 Protective Effects of Fibrinolysis Inhibition on Sepsis‐Induced Lung Injury Exacerbated by *E. faecalis*

**FIGURE 5:**
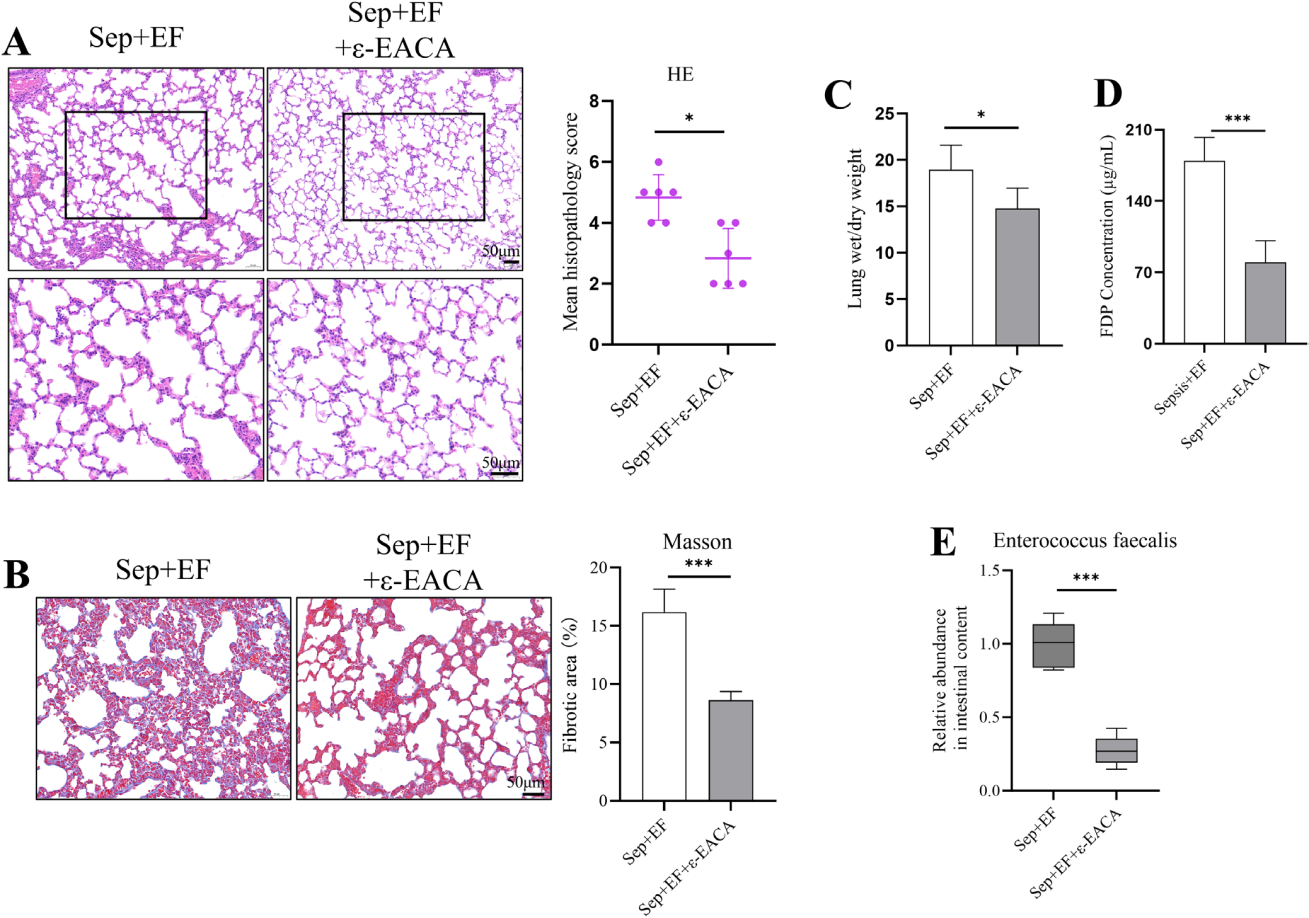
Protective effects of fibrinolysis inhibition on sepsis‐induced lung injury exacerbated by *E. faecalis* . (A) HE staining showed improved lung histology in the ε‐aminocaproic acid (ε‐EACA)‐treated group, with significantly reduced pathological scores compared to the sepsis + *E. faecalis* group. (B) Masson staining revealed reduced collagen deposition following ε‐EACA treatment, mitigating pulmonary fibrosis. (C) Wet‐to‐dry weight ratios demonstrated reduced pulmonary edema in the ε‐EACA‐treated group. (D) FDP levels were significantly decreased with ε‐EACA treatment, indicating reduced fibrinolytic activation. (E) Quantitative analysis showed significantly reduced relative abundance of *E. faecalis* in lung tissues following ε‐EACA treatment. Data are provided as the mean ± SD (*n* = 6 per group). *p < 0.05, ***p < 0.001.

The administration of ε‐aminocaproic acid (ε‐EACA), a fibrinolysis inhibitor, significantly attenuated lung injury in septic mice co‐infected with *E. faecalis* . H&E staining revealed reduced lung injury in the ε‐EACA‐treated group, with significantly lower pathological scores, including decreased alveolar collapse and inflammatory cell infiltration, compared to the sepsis *+ E. faecalis* group (Figure 5A). Masson staining showed that ε‐EACA treatment significantly reduced collagen deposition, indicating mitigation of fibrosis (Figure 5B). The wet‐to‐dry weight ratio, a marker of pulmonary edema, was significantly lower after ε‐EACA treatment (Figure 5C). Furthermore, fibrin degradation product (FDP) levels were markedly decreased following ε‐EACA administration (Figure 5D), suggesting reduced fibrinolytic activation. Notably, quantitative analysis demonstrated that ε‐EACA treatment significantly decreased the relative abundance of *E. faecalis* in lung tissues (Figure 5E), highlighting the potential role of fibrinolysis inhibition in controlling bacterial burden and improving lung pathology.

### 3.6 MitoTEMPO Mitigates Sepsis‐Driven Mitochondrial Damage: ATP Restoration, ROS Reduction, and Ultrastructural Improvement

**FIGURE 6:**
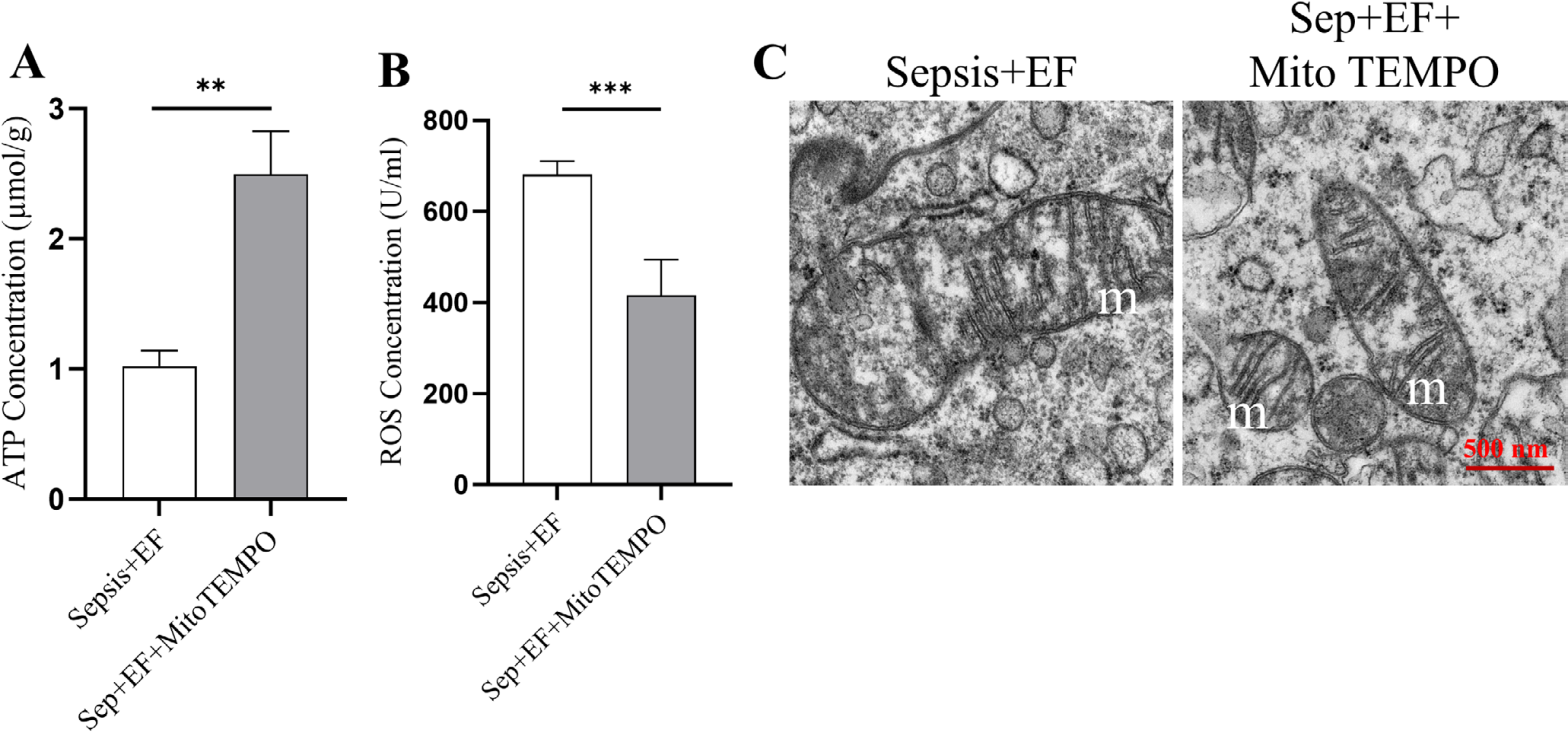
MitoTEMPO mitigates sepsis‐driven mitochondrial damage: ATP restoration, ROS reduction, and ultrastructural improvement. (A) ATP levels were significantly restored in the MitoTEMPO‐treated group compared to untreated septic mice. (B) ROS levels were significantly reduced following MitoTEMPO treatment, reflecting decreased oxidative stress. (C) Electron microscopy images revealed improved mitochondrial morphology in the MitoTEMPO‐treated group, including reduced swelling, rupture, and cristae loss. Data are provided as the mean ± SD (*n* = 6 per group). **p < 0.01, ***p < 0.001.

Treatment with the mitochondrial protector MitoTEMPO significantly improved mitochondrial function and alleviated lung injury in septic mice. Compared to the untreated sepsis group, ATP levels were significantly restored in the MitoTEMPO group (Figure 6A). Additionally, MitoTEMPO treatment reduced ROS levels in lung tissues (Figure 6B), reflecting decreased oxidative stress. Electron microscopy images (Figure 6C) revealed that MitoTEMPO improved mitochondrial morphology, mitigating swelling, rupture, and loss of cristae in septic lung tissues.

### 3.7 Combined Fibrinolysis Inhibition and Mitochondrial Protection Synergistically Alleviate Lung Injury in Sepsis With *E. faecalis* Infection

**FIGURE 7:**
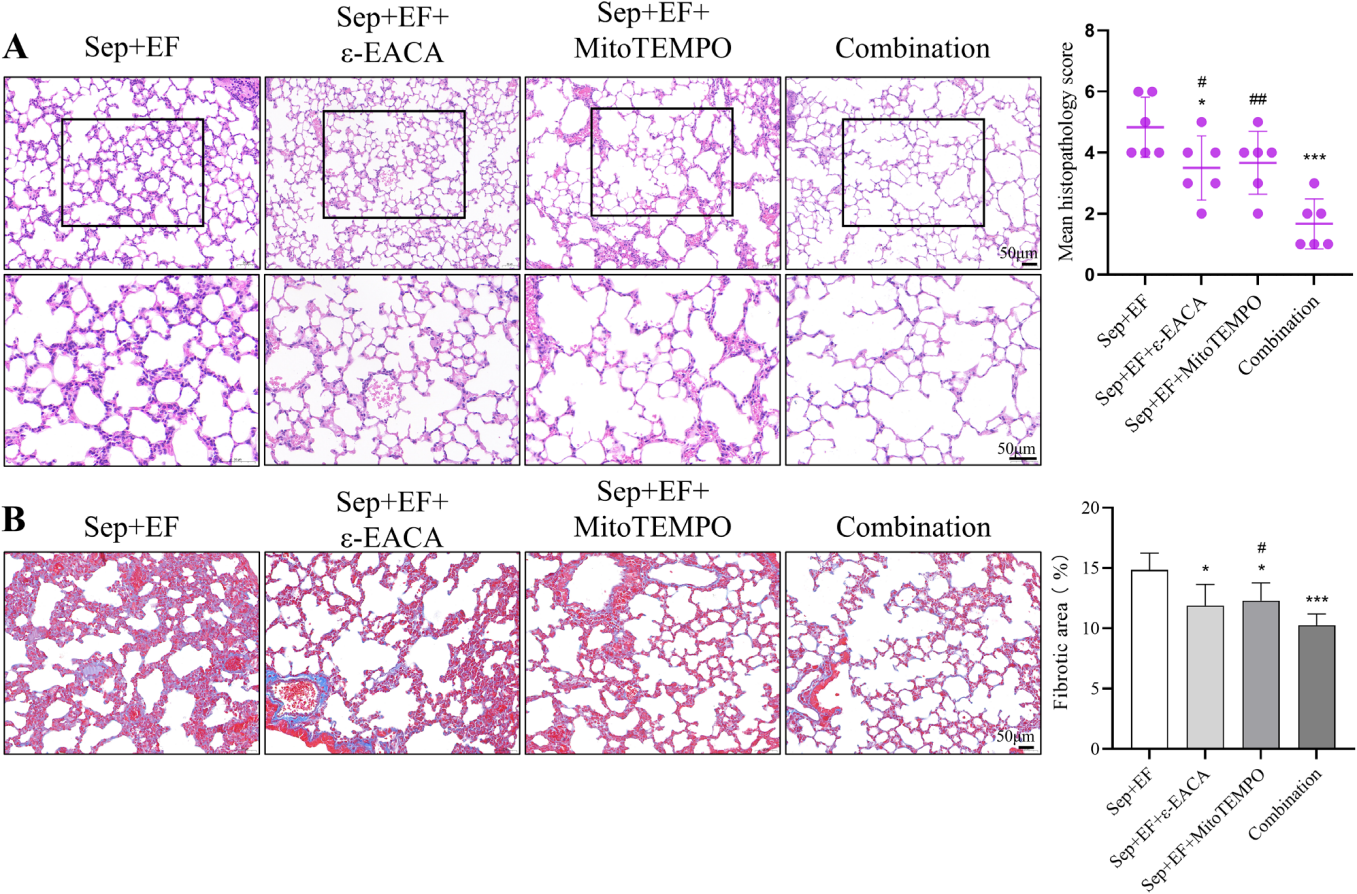
Combined fibrinolysis inhibition and mitochondrial protection synergistically alleviate lung injury in sepsis with *E. faecalis* infection. (A) HE staining demonstrated reduced lung pathology scores in both the ε‐EACA and MitoTEMPO treatment groups, with the combination treatment showing further reductions, indicating a synergistic effect. (B) Masson staining revealed reduced collagen deposition in the ε‐EACA and MitoTEMPO groups, with the combination treatment showing the greatest reduction in fibrosis area. Data are provided as the mean ± SD (*n* = 6 per group). *p < 0.05, ***p < 0.001, #*p* < 0.05, ##*p* < 0.001. *Indicates comparison with the Sep + EF group, ^#^Indicates comparison with the Sep + EF + ε‐EACA group.

The combined treatment of fibrinolysis inhibition (ε‐EACA) and mitochondrial protection (MitoTEMPO) provided enhanced protection against sepsis *+ E. faecalis*‐induced lung injury. H&E staining revealed that both ε‐EACA and MitoTEMPO significantly reduced the pathological scores compared to the sepsis *+ E. faecalis* group, while the combination treatment further reduced the scores, suggesting synergy (Figure 7A). Masson staining demonstrated similar results, with ε‐EACA and MitoTEMPO each reducing the fibrotic area, and the combination treatment showing an even greater reduction in collagen deposition (Figure 7B).

## 4 Discussion

Previous studies have examined *E. faecalis* in hepatocellular carcinoma [30], inflammatory bowel diseases [31], pancreatic cancer [32] and refractory apical periodontitis [33]. In contrast, this study focuses specifically on its contribution to sepsis‐induced lung injury, emphasising the critical interplay between intestinal barrier disruption, microbial translocation, fibrinolytic activation, and mitochondrial dysfunction. Using a CLP model, we observed significant intestinal barrier compromise, evidenced by reduced tight junction proteins and increased permeability, which facilitated the translocation of gut‐derived *E. faecalis* into the lungs. 16S rRNA sequencing and qPCR confirmed *E. faecalis* as a key bacterium involved in lung injury pathogenesis. Further analysis revealed that fibrinolytic system activation promoted extracellular matrix degradation, enabling bacterial colonisation in lung tissues. These processes coincided with mitochondrial dysfunction characterised by oxidative stress and structural abnormalities, which exacerbated pulmonary damage. Collectively, these findings establish a mechanistic link between bacterial translocation, host tissue responses, and organ injury during sepsis, offering potential targets for therapeutic intervention.

Then we explored several key mechanisms involved in the progression of lung damage during sepsis. Our results indicate that the fibrinolytic system plays a central role in facilitating *E. faecalis* translocation to lung tissues through the activation of tissue plasminogen activator (tPA) and plasminogen activator inhibitor‐1 (PAI‐1). By promoting the degradation of extracellular matrix (ECM) components, the system disrupts the lung's structural integrity, facilitating *E. faecalis* colonisation. This finding supports previous studies linking fibrinolysis to tissue invasion and bacterial dissemination in various infectious conditions, such as pneumonia and sepsis. We observed that fibrinolytic system inhibition via a specific tPA inhibitor significantly reduced pulmonary bacterial burden and improved function. These results highlight the therapeutic potential of targeting the fibrinolytic system to mitigate sepsis‐related lung injury. In line with our findings, a study by R. A. Jacobson et al. demonstrated that the application of tranexamic acid (TXA), a PLG inhibitor, can mitigate the effects of *E. faecalis* ‐induced collagenolysis. TXA was found to reduce tissue damage and prevent complications associated with poor intestinal healing.

Beyond fibrinolysis, mitochondrial dysfunction critically exacerbates lung injury. Sepsis‐induced oxidative stress, coupled with mitochondrial structural changes such as shrinkage and disorganised cristae, disrupts cellular energy metabolism. This finding aligns with existing research indicating that mitochondrial dysfunction is a hallmark of sepsis‐induced organ injury, and that mitochondrial damage in the lungs contributes significantly to the pathophysiology of acute lung injury [34, 35]. Notably, mitochondrial protectants like antioxidant agents and targeted therapies, showed promise in mitigating oxidative stress and improving lung outcomes in our model.

Furthermore, the interplay between mitochondrial dysfunction and microbial translocation is a critical factor in sepsis‐induced tissue injury [36]. We found that *E. faecalis* lung translocation exacerbated mitochondrial dysfunction, evidenced by elevated levels of ROS and lipid peroxidation. This suggests a feedback loop, where microbial invasion not only causes inflammation but also triggers cellular damage, thereby worsening tissue injury. Previous studies have suggested that cross‐talk between microbial components and mitochondrial pathways contributes to the systemic inflammatory response observed in sepsis.

Finally, our results elucidate the gut and its role in sepsis pathogenesis. Translocation of gut‐derived bacteria, particularly *E. faecalis* , into the lungs provides new mechanistic insights into how intestinal dysbiosis contributes to sepsis‐induced organ injury. Disruption of the intestinal barrier, facilitated by inflammatory mediators and microbial overgrowth, allows for bacterial translocation, which in turn exacerbates lung inflammation and damage. This phenomenon is consistent with the emerging gut–lung axis, where changes in the gut microbiota influence lung health, and vice versa. Current sepsis therapeutic strategies targeting the fibrinolytic system focus on thrombolytic drugs (such as Antithrombin‐III [37] and Recombinant human soluble thrombomodulin (rhTM) [38]) and antifibrinolytic drugs (such as tranexamic acid [39] and aminocaproic acid [40]). Mitochondrial protection therapy focuses on mitochondrial‐targeted drug delivery to restore function, improve intestinal barrier integrity and alleviate inflammatory responses [41, 42], with ongoing clinical trials of STC3141 [43]. Modulating gut microbiota or reinforcing intestinal barriers could thus yield novel therapies for sepsis‐induced lung injury.

In conclusion, our study highlights the complex interplay of microbial translocation, fibrinolysis, and mitochondrial dysfunction underlying sepsis‐induced lung injury. By elucidating the role of *E. faecalis* and the fibrinolytic system in lung damage, we identify promising therapeutic targets to mitigate sepsis‐related lung injury. However, several limitations warrant consideration. For example, the CLP mouse model, while widely used, may not fully recapitulate the heterogeneity of human sepsis. Besides, our interventions (ε‐EACA and MitoTEMPO) were tested in acute settings; long‐term efficacy and safety in chronic sepsis models require further investigation. Future studies should further explore the therapeutic potential of fibrinolysis inhibitors, mitochondrial protectants, and gut microbiota modulation in improving outcomes for septic patients.

## Author Contributions

**Chenfei Wang:** methodology (equal), validation (equal), writing – original draft (equal). **Dan Lv:** data curation (equal), software (equal). **Yuan Gao:** validation (equal). **Xinhui Xu:** validation (equal). **Changqing Zhu:** validation (equal). **Song Zhang:** writing – original draft (equal). **Keji Zhang:** methodology (equal), writing – review and editing (equal).

## Ethics Statement

All animal experiments were approved by the Ethics Committee of Experimental Animal Welfare of Renji Hospital Affiliated with Shanghai Jiaotong University.

## Consent

The authors have nothing to report.

## Conflicts of Interest

The authors declare no conflicts of interest.

## Data Availability

All data that support the findings in this study are available from the corresponding author upon reasonable request.
